# An Evaluation of the Performance and Economics of Membranes and Separators in Single Chamber Microbial Fuel Cells Treating Domestic Wastewater

**DOI:** 10.1371/journal.pone.0136108

**Published:** 2015-08-25

**Authors:** Beate Christgen, Keith Scott, Jan Dolfing, Ian M. Head, Thomas P. Curtis

**Affiliations:** 1 School of Civil Engineering and Geosciences, Newcastle University, Newcastle upon Tyne, United Kingdom; 2 School of Chemical Engineering and Advanced Materials, Newcastle University, Newcastle upon Tyne, United Kingdom; Purdue University, UNITED STATES

## Abstract

The cost of materials is one of the biggest barriers for wastewater driven microbial fuel cells (MFCs). Many studies use expensive materials with idealistic wastes. Realistically the choice of an ion selective membrane or nonspecific separators must be made in the context of the cost and performance of materials available. Fourteen membranes and separators were characterized for durability, oxygen diffusion and ionic resistance to enable informed membrane selection for reactor tests. Subsequently MFCs were operated in a cost efficient reactor design using Nafion, ethylene tetrafluoroethylene (ETFE) or polyvinylidene fluoride (PVDF) membranes, a nonspecific separator (Rhinohide), and a no-membrane design with a carbon-paper internal gas diffusion cathode. Peak power densities during polarisation, from MFCs using no-membrane, Nafion and ETFE, reached 67, 61 and 59 mWm^-2^, and coulombic efficiencies of 68±11%, 71±12% and 92±6%, respectively. Under 1000Ω, Nafion and ETFE achieved an average power density of 29 mWm^-2^ compared to 24 mWm^-2^ for the membrane-less reactors. Over a hypothetical lifetime of 10 years the generated energy (1 to 2.5 kWhm^-2^) would not be sufficient to offset the costs of any membrane and separator tested.

## Introduction

The material cost is a defining factor in microbial fuel cell (MFC) design as even high power generated does not cover the cost expended on materials used. Focusing on membranes, Nafion, the most commonly used proton exchange membrane (PEM) in microbial fuel cells, is arguably too expensive for use in wastewater treatment [1–6]

The function of the membrane is to separate the anode and cathode reaction in an electrochemical system while permitting selective transport of protons from the anode to the cathode and preventing transport of oxygen into the anode chamber. A porous separator also serves as a barrier separating the anode and cathode reaction but any ions can be transported from the anode chamber to the cathode by diffusion. In choosing a MFC membrane both cost and performance must be considered. A technically superior membrane will only be a rational choice if the putative extra cost is offset by extra performance.

The ideal membrane needs to be selective (proton conducting), durable, chemically stable, biocompatible, resistant to fouling and clogging (especially when using fuels of unknown and changeable composition such as wastewater) and inexpensive. In reality a compromise must be reached between performance and cost. For wastewater treatment low cost, together with durability and resistance to fouling, may be the most important requirement for an economically viable system generating energy even though low oxygen diffusion, ionic resistance and crossover benefit power generation enormously.

The challenges of using membranes and air cathode in a membrane-electrode-assembly (MEA) architecture are: oxygen transport through cathode and membrane into the anode chamber, substrate loss through the membrane, ion transport through the membrane to the cathode where salts (typically carbonates) are formed, resistance to proton transport due to fouling and clogging over time, and cost.

Although Nafion is frequently described as a proton exchange membrane Nafion also transports other cation species (including Na^+^, K^+^, NH^4+^, Ca^2+^, Mg^2+^) found in the anolyte in MFCs [7, 8]. Concentrations of other cations can be 10^5^ times higher than the proton concentration in wastewater which could lead to preferred transfer of other ions through the membrane and salt precipitation on the cathode and inhibition of the cathode catalyst [9–11]. The high permeability to oxygen and substrate (e.g. acetate) of Nafion is also a challenge to the realization of high power densities [2]. Strategies to reduce the cost and overcome these limitations include the use of inexpensive ion and ultrafiltration membranes or separators in a membrane-less MFC design.

Kim *et al*. (2007) reported higher power densities and coulombic efficiencies in MFCs using an anion exchange membrane (AEM) compared to the use of Nafion or an inexpensive cation exchange membrane (CEM). It was suggested that the better performance of the AEM was due to the movement of phosphate or carbonate ions which would also contribute to a better pH balance in the anode and cathode chamber [12].

Membrane-less MFCs use either a non-selective separator as a membrane or no-membrane; i.e. the electrolyte itself acts as barrier between anode and cathode. The most important characteristics of materials used as separators are cost, durability and high mechanical strength. As the separators are not selective, any substance in the substrate can be transferred to the cathode and higher oxygen diffusion into the anode chamber is anticipated. Consequently membrane-less reactors show lower coulombic efficiencies than MFCs with a selective membrane presumably because substrate is digested aerobically [13, 14]. Membrane-less MFCs would be the most cost-effective solution if the challenge of low coulombic efficiencies can be overcome.

The aim of this study was to lay a framework for the evaluation of membranes and separators in microbial fuel cells in terms of cost and performance. For this fourteen plausible low cost membranes and separators were characterised for durability, oxygen diffusion, and ionic resistance. The best performing materials were studied in microbial fuel cell reactors and the performance of the reactor was related to the cost of the membranes.

## Materials and Methods

### 1.1. Membrane and separator materials

The membranes and separators used were

- Nafion 117 (DuPont, USA), a perfluorinated sulphonic acid (PFSA) membrane which has high mechanical strength, chemical stability, high electrical conductivity and selective permeability [8, 15–17] and has generally been used in MFCs as selectively proton conducting membrane.
- Five selective radiation grafted membranes provided by J.A. Horsfall, Cranfield University [18] with good proton conductivity used in fuel cells. All base polymers used were grafted with hydrophilic poly(styrene sulfonic acid) (PSSA). The membranes were:
  ○ ETFE-g-PSSA D.O.G. (degree of grafting) 23% (with a thickness of 125 μm and an ion exchange function (IEC) of 1.784 meq g^-1^)
  ○ ETFE-g-PSSA D.O.G. 35% (50 μm thickness and 1.984 meq g^-1^ IEC);
  ○ PVDF-g-PSSA D.O.G. 34% (30 μm thickness and 2.268 meq g^-1^ IEC);
  ○ CoPVDF-g-PSSA D.O.G. 10% (100 μm thickness and 0.826 meq g^-1^ IEC)
  ○ HDPE-g-PSSA D.O.G. 11% (100 μm thickness and 0.826 meq g^-1^ IEC).
- Rhinohide (RH, Entek International, UK), an inexpensive microporous battery separator that has been used as a cheaper alternative to a selective membrane. RH is a composite of ultra-high-molecular weight polyethylene and silica (UHMWPE/Si) and is characterised by its hydrophilicity, strength and porosity (55±5%).
- Five spunbond nonwoven battery separator materials (Scimat, Freudenberg, Germany), made out of polypropylene (PP) or composite polypropylene/polyethylene (PP/PE) fibres. The surface of the fibres has been UV grafted with polyacrylic acid to increase the hydrophilicity and provide an ion exchange function (around 0.8 meq g^-1^). Scimat 700/30k and 700/40k were neutralised and were in the potassium salt form, while Scimat 700/70, 700/77 and 850/61 were in the acid form. These separators are cheap materials with a high ion exchange function which should lead to good proton conductivity.
- Tyvek (DuPont, USA), a separator made of spun-bonded olefins (high density polytheylene fibres) was chosen due to outstanding chemical and rot and mildew resistance, high porosity and strength.
- Toray carbon paper (E-Tek, UK) was used in a membrane-less MFC (no membrane) as a conductive support for the cathode inside the MFC chamber.

For the radiation grafted membranes an inert polymer base was cross-linked through the use of γ radiation with a monomer forming a grafted copolymer. The radiation grafted membranes have a backbone based on ETFE, PVDF and HDPE incorporating sulphonic acid groups to provide ionic pathways for proton transport [19–21]. The radiation grafting of polymer and monomer with different characteristics means the produced copolymer exhibited the desired hydrophilic properties without impairing the chemical resistance [22].

### 1.2. Material Characterisation

#### 1.2.1. Durability screening

Membranes and separators were immersed in wastewater under anaerobic conditions for a six month period to study biofouling. The wastewater was replaced every two weeks. After six month the membranes were dyed with DAPI and examined under a confocal laser scanning microscope (CLSM; Leica Microsystems Ltd., Milton Keynes, UK) to visualise microorganisms on the surface and especially inside the membrane separators.

#### 1.2.2. Ion conductivity

The ion conductivity was measured in an electrochemical cell with a surface area of 3.2 cm^2^ by measuring the resistance in the cell with a membrane in 1M phosphate buffer solution (PBS) using AC impedance. Impedance measurements were conducted over a frequency range of 30000 to 0.1 Hz with a sinusoidal perturbation of 10 mV amplitude. The background electrolyte resistance R_Electrolyte_ was subtracted from the cell resistance R_Cell_ to obtain the membrane resistance R_Mem_.

$$R_{Mem}=R_{Cell}-R_{Electrolyte}$$ (1)

From the membrane resistance *R*_*Mem*_ the area resistance *R*_*A*_, the resistivity *ρ* and the conductivity *κ* of the membranes were calculated as

$$R_{A}=R_{Mem}∙A$$ (2)

$$\rho =\frac{R_{A}}{L_{t}}$$ (3)

$$\kappa =\frac{L_{t}}{R_{A}}$$ (4)

Where *A* is the membrane area and *L*_*t*_ the membrane thickness [17].

#### 1.2.3. Oxygen mass transfer, diffusion coefficient and membrane permeability

The oxygen permeability of membranes and separators was determined for each membrane using a single chamber microbial fuel cell filled with nitrogen sparged water at 298 K. The anodic chamber was stirred continuously to ensure the same oxygen concentration throughout. A dissolved oxygen (DO) probe was placed in the anode chamber and the DO concentration in the chamber was recorded over time.

In a completely mixed two chamber system, with the chambers separated by a membrane, the mass balance of dissolved oxygen in the chamber is determined through [1, 23]:

$$V\frac{dc}{dt}=J_{O}A=\frac{D_{O}A}{L_{t}}(c_{0}-c)$$ (5)

Where *V* is the chamber volume, *J*_*O*_ is the oxygen flux, *A* is the membrane area, *L*_*t*_ the membrane thickness, *c*_*0*_ is the saturated oxygen concentration in the aerated chamber, *c* is the dissolved oxygen concentration at time *t* in the anode chamber.

The solution of which is

$$D_{O}=-\frac{{VL}_{t}}{At}\ln\;\left[\frac{c_{0}-c}{c_{0}}\right]$$ (6)

Using Eq 6 the diffusion coefficient *D*_*O*_ and the mass transfer coefficient *k*_*O*_ for a two chamber can be calculated as [1, 2]:

$$k_{O}=-\frac{V}{At}\ln\;\left[\frac{c_{0}-c}{c_{0}}\right]$$ (7)

$$D_{O_{2}}=k_{O}L_{t}$$ (8)

In a single chamber microbial fuel cell system the aerated chamber is the air itself. Thus the concentration of oxygen *c*_*0*_ in the air chamber can be determined using Henrys law [24].

$$c_{0}=\frac{k_{H}}{p_{g_{O_{2}}}}$$ (9)

Where *k*_*H*_ is Henry’s coefficient for oxygen and *p*_*gO2*_ is the partial pressure of oxygen. The coefficient *k*_*H*_ = 7.92·10^4^ kPa kg mol^-1^ at 298 K [25].

The membrane permeability *P*_*M*_ was calculated as [26]

$$P_{M}=\frac{k_{Transfer}}{A∙1atm}$$ (10)

Where *k*_*Transfer*_ is the transfer rate of oxygen into the anodic chamber.

### 1.3. Single Chamber Microbial Fuel Cell configuration and operation

**Fig 1:**
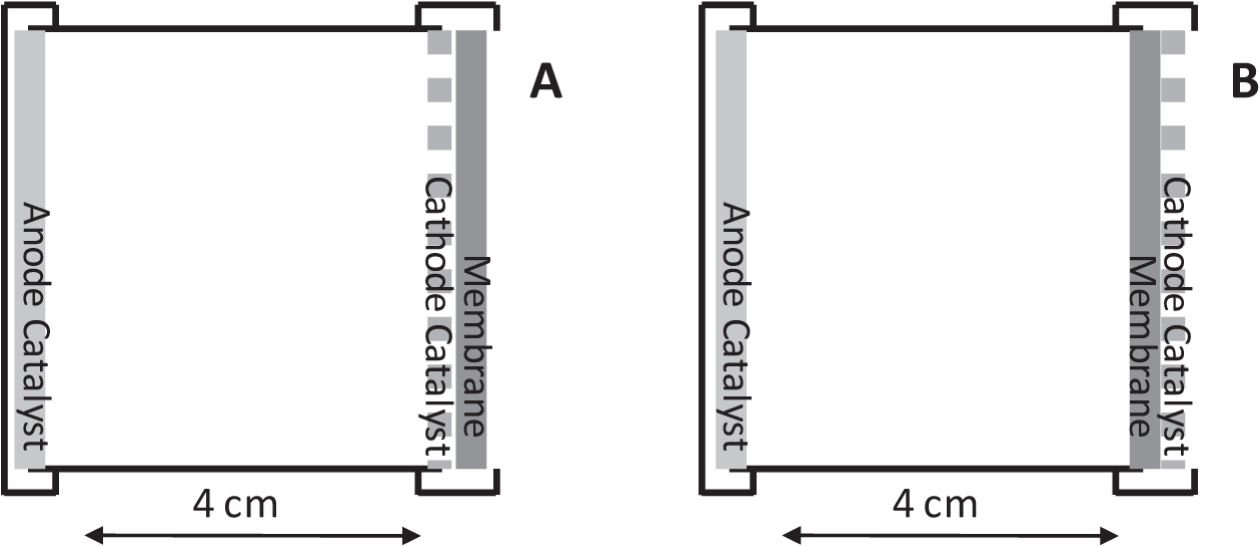
Reactor configurations used during the study with the cathode catalyst applied at the air-side of the membrane (A) or inside the anode chamber (B) for the membrane-less reactor.

Five different membrane materials were studied in a low cost single chamber MFCs using wastewater as a feed and activated carbon cloth (FM30k, Chemviron Carbon, UK) as the anode. The carbon black cathode catalyst was deposited directly onto the membrane material used at a loading of 1 mg cm^-2^. For reactors using Nafion, Rhinohide (RH), ETFE and PVDF as a membrane, the catalyst was applied on the outside of the membrane as an air cathode (Fig 1A). Reactors using carbon paper (no membrane) as membrane-less reactors, had the carbon black catalyst applied on the inside of the chamber opposite to the anode (Fig 1B). A control reactor was run under anaerobic conditions at the same time to simulate anaerobic digestion.

The MFC reactors were made from polyacrylate with a total working volume of 50 cm^3^ (ml) (internal length 4 cm; internal diameter, 12.5 cm^2^). The air cathode (cross sectional area 12.5 cm^2^) was located opposite to the anode in the reactor. The reactors were operated under fed batch mode at room temperature and voltage was monitored continuously using a data acquisition system (ADC 16, Pico Technology Ltd, UK). The substrate (nitrogen purged wastewater) was replaced when the voltage started to decrease. Stabilisation of voltage generation in the reactors indicated acclimatisation of the anodic community and consequent adaptation of system. During acclimatisation and enrichment the reactors were operated under a fixed resistance of 1000 Ω. Reactors were considered acclimatised when the peak voltage was reproducible over consecutive cycles. Once a mature electroactive biofilm covered the anode surface electrochemical (polarisation, linear sweep voltammetry) and analytical (COD, pH, conductivity) measurements were made.

### 1.4. MFC feed

Municipal wastewater was used as feed for the MFCs. Raw wastewater was collected from the influent at a local municipal sewage treatment works (Cramlington Sewage Treatment Works, Northumbrian Water, Cramlington, UK) and kept at 4°C to minimise changes in the wastewater during storage. Wastewater was used as both inoculum and feed. The wastewater used had a COD of 256 mg l^-1^, pH 7.4 and a conductivity of 1.493 mS cm^-1^. The wastewater contained 202 mg l^-1^ suspended solids (SS) and 11% of the SS comprised volatile suspended solids (VSS). 94 mg l^-1^ sulphate and 181 mg l^-1^ chloride were detected in the wastewater. All results shown are the average of two duplicate reactors.

### 1.5. Wastewater characteristics

Suspended solids (SS), volatile suspended solids (VSS) and the chemical oxygen demand (COD) were measured according to Standard Methods for the Examination of Water and Wastewater [27]. Sulphate, phosphate, chloride and nitrate concentrations were estimated by ion-chromatography (DX-100, Dionex International, UK). The ionic conductivity was measured using a handheld conductivity meter pIONeer 30 (Radiometer Analytical, France). The pH was measured using a portable 3310 pH Meter (Jenway, UK) and the dissolved oxygen (DO) was measured using a portable 9500 DO_2_ Meter (Jenway, UK).

The coulombic efficiency (*CE*) was calculated using [28]

$$CE=\frac{M\int_{0}^{t}Idt}{FbV_{Anode}\Delta COD}$$ (11)

Where *M* is the molecular weight of oxygen, *F* is faraday’s constant in coulomb, *I* the current in ampere, *b* = 4 is the number of electrons exchanged per mole of oxygen, *V*_*Anode*_ is the liquid volume of the anode chamber, and *ΔCOD* is the change in COD over time.

### 1.6. Electrochemical measurement

During a feeding cycle, samples were taken for COD, pH and ion chromatographic measurements. Following this, the reactors were refilled at the open circuit potential (OCP). Once the voltage stabilised, the reactors were polarised. During polarisation the change in cell current and voltage, as well as the anode and cathode potentials under polarisation (vs. a Ag/AgCl reference electrode), were recorded continuously using a data acquisition system (ADC 16, Pico Technology Ltd, UK). Polarization curves were recorded starting at OCP using a potentiostat (GillAC, ACM Instruments, UK) at a scan rate of 1 mV s^-1^.

Anode and cathode potentials were monitored during cell polarisation using an Ag/AgCl reference electrode (Thermo Scientific, UK) placed in the anode chamber through a capillary using phosphate buffer (100 mM; pH 6.5) as electrolyte. The cathode potential were internal resistance (iR) corrected as the cathode potential had to be measured through the membrane.

The internal resistance was measured by electrochemical impedance spectroscopy using a potentiostat (Gillac, ACM Instruments). Impedance measurements were conducted at OCP over a frequency range of 30000 to 0.1 Hz with a sinusoidal perturbation of 10 mV amplitude.

## Results and Discussion

### 1.7. Membrane and separator characteristics

Thirteen low cost membranes and separators were characterised for durability, oxygen diffusion and ionic resistance. Additionally Nafion was characterized for comparison and together with the best performing low cost separators and membranes was subsequently tested in microbial fuel cell reactors.

Nine materials out of the fourteen studied were mechanically unsuitable as MFC membranes. The five Scimat materials leaked during conductivity and oxygen diffusion measurements. Tyvek and HDPE both exhibited very high resistivity and area resistance (S1 Table) and the highly grafted membranes ETFE 35% and PVDF 34% exhibited high levels of warping and swelling which made them unsuitable for use in MFC reactors.

The remaining membrane and separator materials exhibited no visible degradation or accumulation of bacteria in the materials after six month immersion in wastewater when observed under a confocal laser scanning microscope. The Rhinohide separator showed biofilm growth on the surface of the material but no degradation was visible on the material itself.

Of the remaining five materials Rhinohide (RH) showed the highest conductivity followed by ETFE, Nafion and PVDF. ETFE showed the lowest area resistance followed by Rhinohide, Nafion and PVDF (S1 Table). Comparisons with published literature are difficult as few studies investigated the membrane resistance in MFCs and in those few reported experimental conditions as well as the membranes investigated differed widely from the conditions applied in this study; thus direct comparisons are not useful.

Similar mass transfer coefficients (k_O_) (around 2.6·10^−3^ cm s^-1^) were obtained for all membrane separators with the exception of carbon paper (3.73 10^−3^ cm s^-1^) (S2 Table). The oxygen diffusion permeabilities observed in this study (5.1 10^−5^ cm^2^ s^-1^ for Nafion) were ten times higher than values reported in literature for Nafion (5.27 10^−6^ cm^2^ s^-1^ and 2.4 10^−6^ cm^2^ s^-1^) presumably due to different electrolytes used in the different studies (water instead of glucose or acetate with PBS) [1, 13, 29, 30].

Based on durability, conductivity, ion exchange capability and oxygen diffusion permeabilities the ion exchange membranes Nafion, ETFE 23% grafted (ETFE) and PVDF 10% grafted (PVDF) and separators Rhinohide (RH) and carbon paper (no-membrane) were selected for evaluation in MFCs. Carbon paper was used as gas diffusion cathode support in a no-membrane design.

### 1.8. MFC studies

#### 1.8.1. Variations of voltage evolution under 1kΩ load

**Fig 2:**
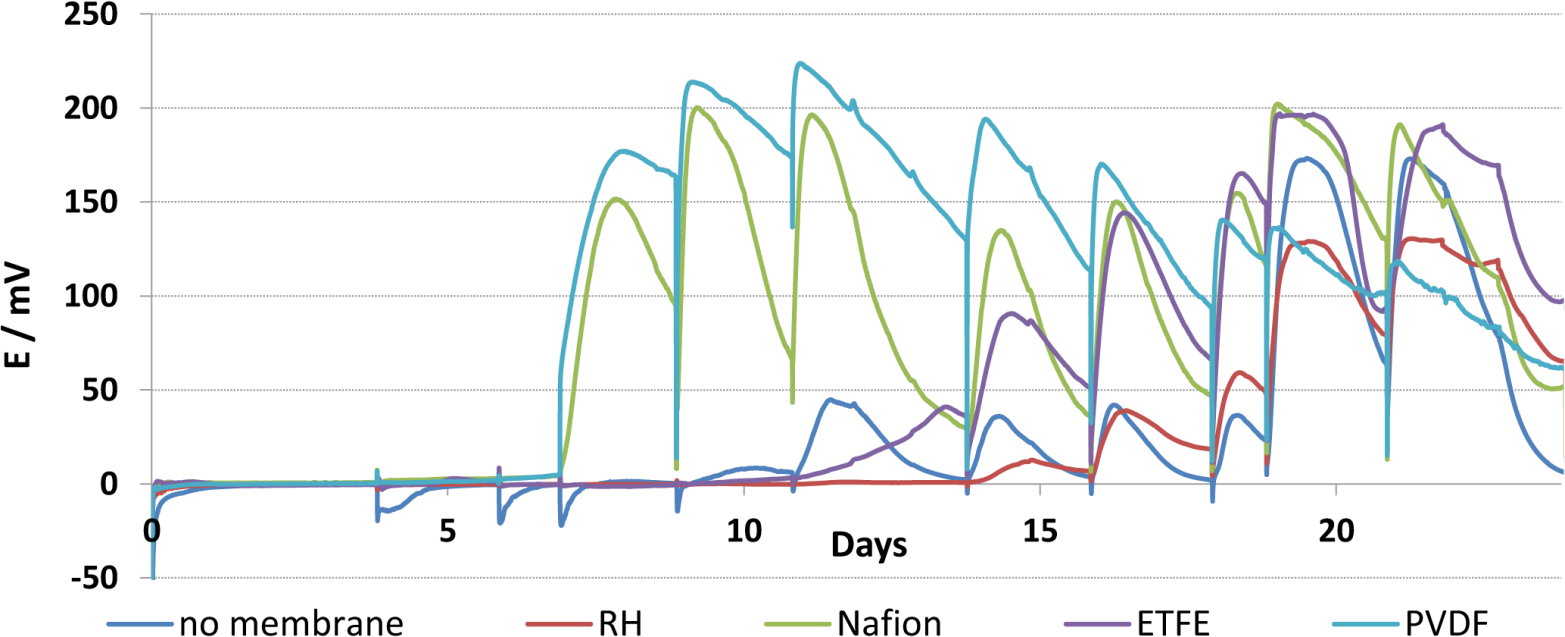
Voltage generation under 1 kΩ load for the different membrane separators. The mean voltage of duplicate reactors is shown.

Variations in acclimatisation time and peak voltage between reactors with different membranes showed the significant influence of the membrane on the microbial fuel cell system (Fig 2). MFC reactors using Nafion and PVDF membranes showed the highest voltage production after 7 days. Both membrane materials showed high area resistances but also low oxygen diffusion coefficients during the material characterization. This suggests that the area resistance or conductivity of the materials does not correlate with performance in MFCs later whereas the oxygen diffusion coefficient could be a good indicator for power production in single chamber MFCs. As oxygen is a competing terminal electron acceptor to the anode it is well known that oxygen diffusion into the anode chamber reduces power generation [2, 13]. The internal resistance is known to affect power and varied from a minimum of 269±4 Ω for Nafion, 294±5 Ω for ETFE, 301±9 Ω for carbon paper and 316±2 Ω for PVDF to a maximum of 350±6 Ω for Rhinohide.

#### 1.8.2. Polarisation studies

**Fig 3:**
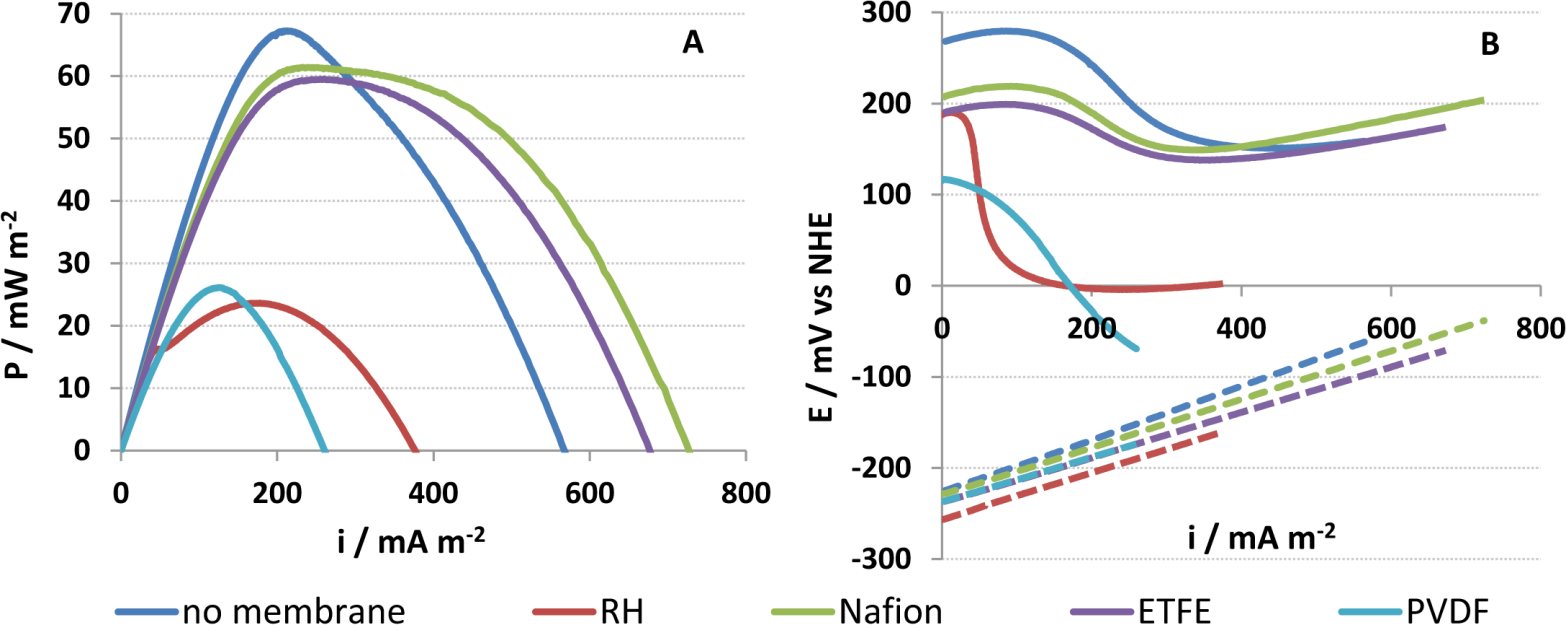
Linear sweep voltammograms showing the peak power density (A) and the iR corrected electrode potential (B) with the anode (dashed) and cathode (line) potential for the reactors using different membranes during polarisation.

Potential, current and power density, achieved during polarisation, are indicators for the performance of a material. High power densities during polarisation were obtained for reactors using no membrane, Nafion and ETFE (Fig 3A). The lowest power densities were observed using Rhinohide and PVDF. One reason for the high power achieved during polarization for Nafion and ETFE could be the low area resistance coupled with a low oxygen diffusion coefficient for these materials. The MFC reactor with no membrane showed high potential power as well. This could be due to the absence of a diffusion rate limiting membrane. The coefficient of variation in peak power density for reactors with no membrane was 30% higher than for reactors with membranes (3% to 10%). The higher coefficient in the no membrane reactor could be due to a higher variability in the uptake of oxygen on the cathode and thus the oxygen concentration in the anode chamber.

#### 1.8.3. Anode and cathode behavior

The anode showed linear variation between overpotential and current density with a typical voltage loss of 100 mV over 400 mA m^-2^ and no significant variation in onset potential for the different reactors (Fig 3B). The cathode behaviour varied widely with the different membrane materials. The anode was limiting in the better performing reactors (carbon paper, Nafion, ETFE) as higher overpotential losses were observed on the anode than the cathode over the whole current density range although carbon black was used as cathode catalyst. The poor performing reactors (PVDF and Rhinohide) showed higher overpotential losses on the cathode than the anode showing that the cathode was more limiting.

The carbon black cathodes showed a two wave profile (Fig 3B), presumably reflecting a change in oxygen reduction kinetics or mechanism [31, 32]. Changes in overpotential may be associated with changes in local cathode pH and with formation of peroxide as part of a two electron mechanism for oxygen reduction using carbon materials [33]. The curvature of the cell potential mirrored the cathode potential (Fig 3B). This showed that the cathode behaviour was the main influence on the system presumably through the influence of the different membranes on the cathode performance. The reactors using better performing membrane separators, carbon paper, ETFE and Nafion showed a wide curved profile in potential with increasing current density while Rhinohide showed a rapid decrease after the first wave before leading into the second wave. The profile of the reactor using the PVDF membrane showed little curvature and only the first wave was clearly visible.

#### 1.8.4. Variations in steady state power density

**Fig 4:**
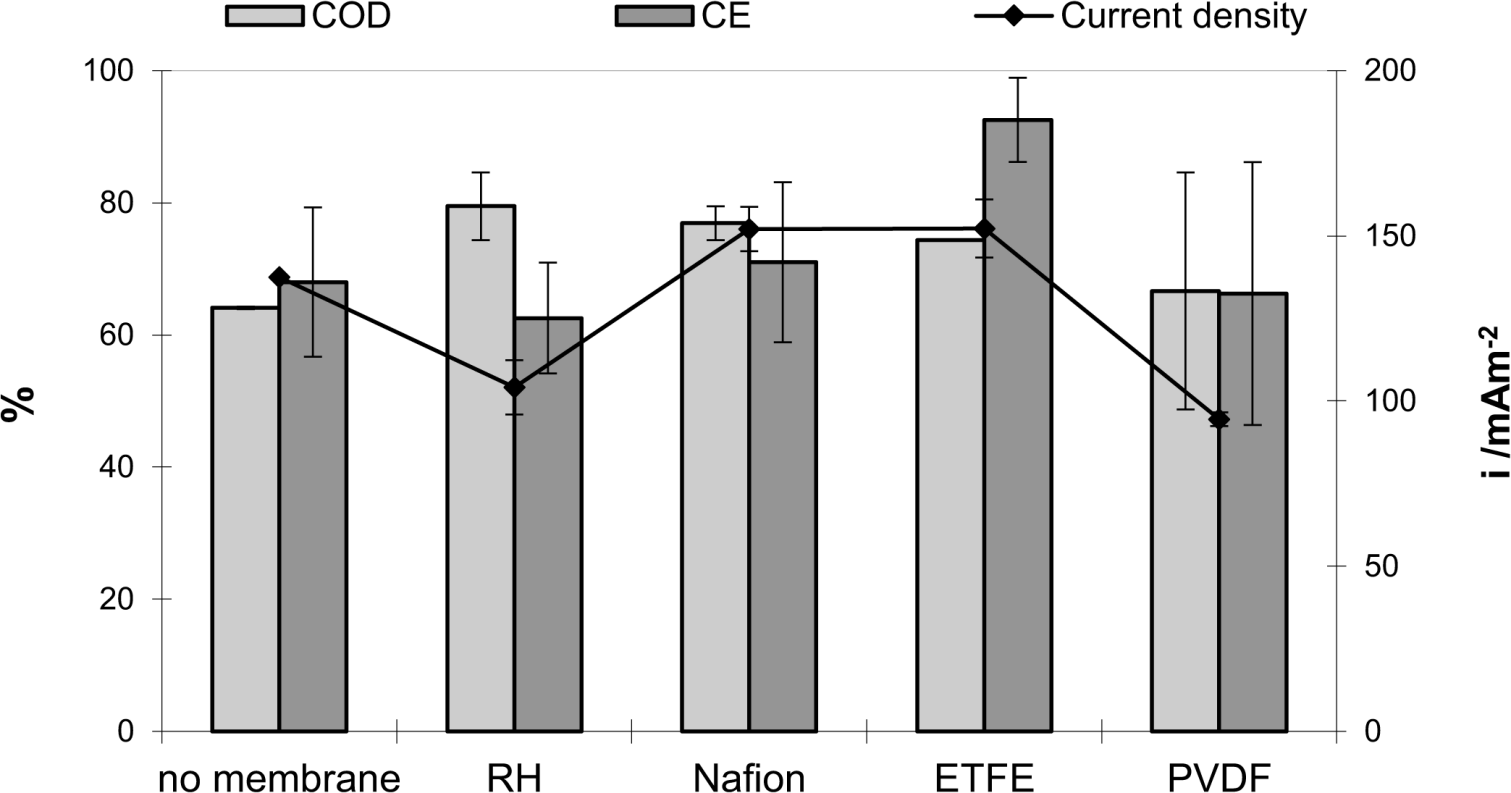
COD removal, coulombic efficiency and current density in wastewater fed MFCs equipped with different membranes. The current density was measured at the end of the batch under 1kΩ external load.

Reactors using ETFE and Nafion membranes achieved the highest steady state power densities with 29±3.4 mW m^-2^ (at 152±8.8 mA m^-2^ and 190±11 mV) and 29±2.6 mW m^-2^ (at 152±6.7 mA m^-2^ and 190±8 mV) respectively under 1kΩ load. The power density in reactors using no membrane reached 24±0.02 mW m^-2^ (at 138±0.07 mA m^-2^ and 172±0.09 mV) and reactors using Rhinohide separators achieved power densities of 14±2.2 mW m^-2^ (Fig 4). The worst performing reactors reaching power densities of 11±0.5 mW m^-2^ used PVDF membranes. The different membrane materials had a significant effect on the cell voltage (p = 0.008), power density (p = 0.010) and current density (p = 0.010). The duplicates were statistically indistinguishable.

Three to five times higher power densities (of 146 mW m^-2^ [13] and 91 mW m^-2^ [34]) were achieved in other studies with wastewater as substrate, platinum as cathode material and no membrane. Nevertheless, power densities reported here are significant and encouraging as they were generated using inexpensive carbon black, without a rare earth catalyst, as cathode material which was not optimized.

#### 1.8.5. Wastewater treatment efficiency

High coulombic efficiencies for wastewater as substrate were observed for all reactors in this study (Fig 4). The high coulombic efficiencies maybe attributed to the use of activated carbon cloth as anode material. However we cannot be sure about this unless and until this has been studied in detail.

The CE observed was similar to those seen by Lorenzo *et al*. (2009) with 63% CE using domestic wastewater as substrate in a continuous flow reactor at very low influent COD (55 mg l^-1^)[35]. Another study reported 27% CE with wastewater as substrate using a continuous flow design with reduced electrode spacing at similar influent COD to this study but a very low flow rate [36]. As both studies used continuous flow reactors in different ways and used very low influent COD it is difficult to relate the results directly to this study.

#### 1.8.6. Cost vs. performance

Energy contained in wastewater is a valuable resource [37], but the use of costly materials to harness this energy is only economical if the power generated covers the added costs (Table 1). In this study that is not the case for the materials tested. The cost of the membranes was estimated using producer pricing for no membrane (CP), Rhinohide and Nafion (Table 1). For the radiation grafted membranes ETFE and PVDF the cost of the base film was multiplied by 10 to estimate the cost of the final membrane. The total power generated over an assumed lifetime of 10 years was calculated as

Energygeneratedover10years=P∙t (12)

where *P* is the kW m^-2^ and *t* is the time (10 years), assuming that the average peak power achieved under 1000 **Ω** in the batch reactors will be stable over their lifetime. Over the assumed lifetime of 10 years the reactor using the comparatively best performing membrane ETFE would produce 2.5 kWh m^-2^ of membrane material generating a total income of 0.18 € m^-2^. This would not cover the capital cost of even the inexpensive membrane at 2.65 € m^-2^ (Table 1). None of the membranes and separators tested in this study would produce enough power to cover the extra cost. Five and ten times higher power generated in other studies [13, 34, 38] would not begin to cover the extra cost either.

**Table 1:** Cost-performance ratio for the different membrane materials. Cost was linked to power density.

	Cost / € m^-2^	P / mW m^-2^	Cost/P / € mW^-1^	Energy generated over a lifetime of 10 years / kWh m^-2^	Total income generated over 10 years / €^a^ m^-2^
**No membrane**	93	24±0.02	3.93	2.1	0.15
**Rhinohide**	1.3	14±2.2	0.10	1.2	0.08
**Nafion**	448	29±2.6	15.5	2.5	0.18
**ETFE**	2.7	29±3.4	0.09	2.5	0.18
**PVDF**	1.8	11±0.5	0.16	1	0.07

^a^At the current electricity selling price in the UK of 0.07 € kWh^-1^.

Predicting technical demands and achievements is difficult. Rozendal *et al*. (2008) in their assessment of the economics of microbial fuels cells for example used a future costs for membranes of 5 € m^-2^ in 2008 while our estimates (Table 1) are already well below this value [4]. It is clear nevertheless that power output needs to improve many fold from the present values of 30 mW m^-2^ (this work) to 150 mW m^-2^ or 240 mW m^-2^ [34, 38] using wastewater as substrate to make this technology even begin to pay for the costs of membranes, let alone for other probably more expensive items such as anode, cathode and current collector. Fitting this all into a compact design is an additional challenge. At the present time the use of MFCs as a wastewater treatment technology is only realistic if value is added through the production of value added products such as hydrogen or ethanol or as a power source for microelectronic devices. However added production steps will always add their own technological limitations to the system.

## Conclusions

Of the fourteen membrane and separator materials characterised three membrane materials (Nafion and radiation grafted membranes based on ETFE and PVDF), one nonspecific separator (Rhinohide), and a no-membrane design with a carbon-paper internal gas diffusion cathode were tested in a low cost reactor design.

Nafion, ETFE and the no membrane design performed best and achieved very high coulombic efficiencies. But the generated electricity, if sold, would not cover the cost of the membranes over a hypothetical lifetime of 10 years, though value added products or the use of an MFC as a power source for microelectronic devices might offer economic value.

A major advantage of MFCs is direct generation of energy. Most technological developments (e.g. batteries) took decades or more to mature. Although various recent studies have resulted in a much deeper understanding of limitations and challenges in MFCs, many more studies with real wastewater as substrate will be needed to develop low cost, efficient components and reactor designs leading to commercial use of MFCs for wastewater treatment in the future.

## Supporting Information

S1 Table Conductivity measurements of the separators and membranes studied using AC impedance spectroscopy in 1M PBS. (PDF)

S2 Table Oxygen diffusion, mass transport coefficient and permeability of the membrane separators in water at 298 K.^a^
(PDF)
