# Supplementary material for: An Evaluation of the Performance and Economics of Membranes and Separators in Single Chamber Microbial Fuel Cells Treating Domestic Wastewater
Source: PLoS One. 2015 Aug 25;10(8):e0136108. doi: 10.1371/journal.pone.0136108 (PMC4548946; doi:10.1371/journal.pone.0136108)
Supplement: S1 Table — (PDF) [file pone.0136108.s001.pdf]

S1 Table. Conductivity measurements of the separators and membranes studied using AC impedance spectroscopy in 1M PBS.

Membrane or separator	Thickness / µm	Area resistance / Ω cm^2	Conductivity / 10^{-3} S cm^{-1}	Resistivity / Ω cm
**Nafion**	183	1.93	9.5	105.65
**ETFE-g-PSSA D.O.G. 23%**	164	1.05	15.7	63.84
**ETFE-g-PSSA D.O.G. 35%**	66	0.48	13.8	72.32
**HDPE-g-PSSA D.O.G. 11%**	39	6.77	0.6	1734.70
**CoPVDF-g-PSSA D.O.G. 10%**	115	3.53	3.3	307.25
**PVDF-g-PSSA D.O.G. 34%**	45	0.49	9.1	109.63
**Rhinohide**	668	1.69	39.4	25.35
**Scimat 700/70**	127	0.49	25.7	38.85
**Scimat 700/77**	117	0.51	23.0·	43.53
**Scimat 700/30k**	130	0.27	48.3	20.72
**Scimat 700/40k**	103	0.78	13.2	75.86
**Scimat 850/61**	91	1.07	8.5	117.51
**Tyvek**	146	31.09	0.5	2129.13
**Carbon paper**	300	n/a	n/a	n/a
