# Supplementary material for: An Evaluation of the Performance and Economics of Membranes and Separators in Single Chamber Microbial Fuel Cells Treating Domestic Wastewater
Source: PLoS One. 2015 Aug 25;10(8):e0136108. doi: 10.1371/journal.pone.0136108 (PMC4548946; doi:10.1371/journal.pone.0136108)
Supplement: S2 Table — (PDF) [file pone.0136108.s002.pdf]

S2 Table. Oxygen diffusion, mass transport coefficient and permeability of the membrane separators in water at 298 K.[a]

Membrane or separator	k_O / 10^{-3} cm s^{-1}	D_O /10^{-5} cm^2 s^{-1}	P_M / 10^{-6} mg l^{-1} s^{-1} cm^{-2} Pa
**Nafion**	2.79	5.1	4.38
**Rhinohide**	2.44	16.3	4.52
**Tyvek**	2.62	3.82	4.61
**Carbon paper (wet proofed)**	3.73	11.2	8.62
**ETFE-g-PSSA D.O.G. 23%**	2.54	4.16	5.01
**ETFE-g-PSSA D.O.G. 35%**	2.17	1.43	8.2
**HDPE-g-PSSA D.O.G. 11%**	2.46	0.957	4.83
**PVDF Copolymer-g-PSSA D.O.G. 10%**	2.79	3.2	4.24
**PVDF-g-PSSA D.O.G. 34%**	2.74	1.23	4.25

[a] None of the Scimat separators could be analyzed due to water leakage.
